# (Ultra-)low dosing of rituximab in rheumatoid arthritis: chances and challenges

**DOI:** 10.1093/rap/rkab007

**Published:** 2021-02-04

**Authors:** Alfons A den Broeder, Nathan den Broeder, Lise M Verhoef

**Affiliations:** Department of Rheumatology, Sint Maartenskliniek, Ubbergen, The Netherlands

**This editorial refers to ‘Low-dose rituximab protocol in rheumatoid arthritis—outcome and economic impact’, by Parvathypriya Chandramohan *et al.***

In a recent paper in this Journal, Chandramohan *et al.* [1] showed, in an uncontrolled study, that a single infusion of 500 mg rituximab (RTX) can be effective in a large proportion of RA patients. At face value, it seems rather peculiar that 14 years after registration of RTX for RA in the USA (1 March 2006) the evidence for optimal RTX dosing still keeps evolving, and this deserves a closer look.

Rituximab, a biological DMARD targeting CD20^+^ B cells, effectively decreases disease activity in patients with RA and can prevent disease progression. The current recommendation of the US Food and Drug Administration and European Medicines Agency consists of two doses of 1000 mg RTX (2 weeks apart), every 6 months. RTX had originally been developed as a treatment for non-Hodgkin’s lymphoma, and the dosing for patients with RA was based directly on dosing used in lymphoma.

Interestingly, no classical phase I/II dose finding studies have been done in RA. This is likely to be attributable to choices by the originator pharmaceutical company, Roche, presumably based on the apparent safety of the higher 4 × 1000 mg dosing and a relative lack of interest in developing this drug in RA. There was also, potentially, a lack of incentive to study lower dosing of RTX in RA, because the price for non-Hodgkin’s lymphoma had already been established, and different cross-disease pricing is cumbersome, if not unfeasible. Thus, a (much) lower RTX dose for RA would be unattractive commercially.

To our knowledge (after searching patent documentation, published trials and trial registries and contacting Roche) the above factors resulted in a lack of formal clinical testing of doses lower than 1 × 1000/2 × 500 mg, with the exception of a very small study combining 500 mg/m^2^ RTX (i.e. ∼850 mg with an average body surface area) with CYC in four patients [2]. This knowledge gap has been identified by several researchers, and since the early 2010s a number of studies have been done in a kind of race to the bottom regarding optimal RTX dosing in RA.

First, it was shown successfully that the effects of RTX are comparable when using 1 × 1000 mg/2 × 500 mg compared with the authorized 2 × 1000 mg in three randomized controlled trials [3]. But things would not end here. In three case reports and a small observational open label study, even (much) lower doses of RTX (50–200 mg) led to complete peripheral B cell depletion and often apparently adequate disease control [4].

Based on these serendipitous findings, the REDO (https://www.thelancet.com/journals/lanrhe/article/PIIS2665-9913(19)30066-9/fulltext) study was designed, showing that in RA patients doing well on RTX 1 × 1000 mg/2 × 500 mg, a retreatment strategy with 1 × 500 or 1 × 200 mg was as effective, although the study failed formally to meet the primary per protocol non-inferiority outcome [4]. Interestingly, the 200 mg seemed to work at least as well (pharmacokinetically, pharmacodynamically and clinically) as 500 mg, and this again suggests that even lower dosing might be possible.

Now comes the study by Chandramohan *et al.* [1], showing the apparent effectiveness of 1 × 500 mg RTX in RA patients commencing RTX treatment. This study has some important limitations, including absence of a control group and the open label design, with suggestion of some regression to the mean in disease activity measurements, in addition to intensive co-treatment with conventional synthetic DMARDs. However, in our view the data still strongly suggest that 1 × 500 mg is an effective treatment in RA, and this notion fits in well with the aforementioned established evidence base.

Some questions remain. How low can or should we go? Why even bother? Regarding the first question, no clinical dose–response curve for RTX in RA has ever been published that clearly shows a relationship plateauing off at a certain dose. Indeed, complete peripheral B cell depletion has been demonstrated after dosages even as low as 1 or 3 mg. Therefore, the jury is still out on this, and it might well be that even (much) lower dosages than 200 mg, albeit maybe combined with a treatment interval shorter than 6 months, are enough to treat RA. But why pursue this? After all, RTX already seems very safe in the higher dosages, and costs seem manageable, especially when using standard low dosing of 1 × 1000 mg/2 × 500 mg and biosimilars where available. We think that the main drivers for researching the lowest effective RTX dose in RA are threefold: safety, cost and ease of use.

Regarding safety, although RTX has a low infection risk associated with it [5], the REDO study showed that infections were 50% lower for the 500 and 200 mg compared with 1000 mg dosages, even in this relatively small study. Also, RTX seems to be a risk factor for specific infections, such as JC virus (the causative agent of progressive multifocal leukoencephalopathy) [6] and, more recently, potentially coronavirus disease 2019 (COVID-19) [7]. These risks have, however, been estimated in real-world data, using full- or half-dosed RTX, thus the use of lower dosages might well ameliorate these already small risks further. Also of interest in these times of COVID-19 are data showing that humoral vaccine response is decreased after RTX. Although there is not yet supporting evidence for this, conceptually, the use of very low-dosed RTX might well ameliorate this disadvantageous effect.

Concerning cost, it should be noted that the view of non-high-income countries on what costs are acceptable differs vastly from that of high-income countries, as mentioned explicitly by Chandramohan *et al.* [1]. Even in The Netherlands, lowering the dose per administration from 1 × 1000 mg per 6 months to 1 × 500 mg would halve the net yearly RTX cost to €2300 ($2600); considerably lower, but still six times more costly than oral MTX. Also, infusion facilities add costs to the total cost of RTX treatment, and this observation also leads to the third reason to go as low as we can: ease of use.

Finally, lower doses can greatly improve the ease of use by opening the possibility of s.c. administration. An s.c. administration of RTX is being used in haematology, but includes a rather large volume of >11 ml. Keeping a bioavailability of 70% in mind, 200 mg i.v. dosing might be substituted elegantly for ∼300 mg s.c. RTX dosing (∼2 ml), negating the need for infusion facilities and reducing some patient burden associated with i.v. administration [8].

In conclusion, we think that the study by Chandramohan *et al.* [1] highlights the road we have travelled regarding RTX dosing in RA, but also illuminates the road ahead with lower doses of RTX, potentially leading to the safest and most cost-effective modern treatment option in RA.

*Funding*: No specific funding was received from any bodies in the public, commercial or not-for-profit sectors to carry out the work described in this manuscript.

*Disclosure statement*: A.dB. has received consultancy honoraria and congress invitations and research grants (to the institution) from Abbvie, Amgen, Cellgene, Roche, Biogen, Lilly, Novartis, Celltrion, Sanofi and Gilead. A.dB. and N.dB. are co-inventors on an RTX-related patent (pending). L.M.V. has declared no conflicts of interest.
